# No need for secondary *Pneumocystis jirovecii* pneumonia prophylaxis in adult people living with HIV from Europe on ART with suppressed viraemia and a CD4 cell count greater than 100 cells/µL

**DOI:** 10.1002/jia2.25726

**Published:** 2021-06-12

**Authors:** Andrew Atkinson, Jose M. Miro, Amanda Mocroft, Peter Reiss, Ole Kirk, Philippe Morlat, Jade Ghosn, Christoph Stephan, Cristina Mussini, Anastasia Antoniadou, Katja Doerholt, Enrico Girardi, Stéphane De Wit, David Kraus, Marcel Zwahlen, Hansjakob Furrer, the Opportunistic Infections Working Group of the Collaboration of Observational HIV Epidemiological Research Europe (COHERE) study in EuroCOORD, Stephane De Wit, A. Antoniadou, A Castagna, K Doerholt, G Fätkenheuer, D Raben, R Teira, R Zangerle, Ali Judd, Robert Zangerle, Giota Touloumi, Josiane Warszawski, Laurence Meyer, François Dabis, Murielle Mary Krause, Catherine Leport, Linda Wittkop, Ferdinand Wit, Maria Prins, Heiner Bucher, Diana Gibb, Gerd Fätkenheuer, Julia Del Amo, Niels Obel, Claire Thorne, Santiago Pérez‐Hoyos, Osamah Hamouda, Barbara Bartmeyer, Nikoloz Chkhartishvili, Antoni Noguera‐Julian, Andrea Antinori, Antonella d’Arminio Monforte, Norbert Brockmeyer, Luis Prieto, Pablo Rojo Conejo, Antoni Soriano‐Arandes, Manuel Battegay, Roger Kouyos, Jordi Casabona, Antonella Castagna, Tessa Goetghebuer, Anders Sönnerborg, Carlo Torti, Caroline Sabin, Ramon Teira, Myriam Garrido, David Haerry, Dominique Costagliola, Antonella d’Arminio‐Monforte, Antonella Castagna, Julia del Amo, Dorthe Raben, Geneviève Chêne, Ali Judd, Pablo Rojo Conejo, Diana Barger, Christine Schwimmer, Monique Termote, Linda Wittkop, Casper M Frederiksen, Dorthe Raben, Rikke Salbøl Brandt, Juan Berenguer, Julia Bohlius, Vincent Bouteloup, Heiner Bucher, Alessandro Cozzi‐Lepri, François Dabis, Antonella d’Arminio Monforte, Mary‐Anne Davies, Julia del Amo, Maria Dorrucci, David Dunn, Matthias Egger, Marguerite Guiguet, Sophie Grabar, Ali Judd, Olivier Lambotte, Valériane Leroy, Sara Lodi, Sophie Matheron, Laurence Meyer, Susana Monge, Fumiyo Nakagawa, Roger Paredes, Andrew Phillips, Massimo Puoti, Eliane Rohner, Michael Schomaker, Colette Smit, Jonathan Sterne, Rodolphe Thiebaut, Claire Thorne, Carlo Torti, Marc van der Valk, Linda Wittkop

**Affiliations:** ^1^ Department of Infectious Diseases Bern University Hospital Inselspital University of Bern Bern Switzerland; ^2^ Infectious Diseases Service Hospital Clinic – IDIBAPS University of Barcelona Barcelona Spain; ^3^ Centre for Clinical Research Epidemiology, Modelling and Evaluation (CREME) Institute for Global Health UCL London UK; ^4^ Department of Global Health Amsterdam University Medical Centers University of Amsterdam Amsterdam The Netherlands; ^5^ Amsterdam Institute for Global Health and Development, and HIV Monitoring Foundation Amsterdam The Netherlands; ^6^ CHIP Department of Infectious Diseases, Rigshospitalet University of Copenhagen Copenhagen Denmark; ^7^ Internal Medicine and Infectious Diseases Department University Hospital of Bordeaux Bordeaux France; ^8^ Service des Maladies Infectieuses et Tropicales Groupe Hospitalier Universitaire Bichat‐Claude Bernard Paris France; ^9^ INSERM U 1137 IAME Université de Paris Paris France; ^10^ Infectious Diseases Unit at Medical Center no.2 Frankfurt University Hospital Goethe University Frankfurt Germany; ^11^ Clinic of Infectious Diseases University of Modena and Reggio Emilia Modena Italy; ^12^ Fourth Department of Internal Medicine ATTIKON University Hospital National and Kapodistrian University of Athens Athens Greece; ^13^ Paediatric Infectious Diseases Unit St. George’s University Hospital London UK; ^14^ Clinical Epidemiology Unit National Institute for Infectious Diseases L. Spallanzani‐IRCCS Rome Italy; ^15^ Department of Infectious Diseases St Pierre University Hospital Université Libre de Bruxelles Brussels Belgium; ^16^ Department of Mathematics and Statistics Masaryk University Brno Czech Republic; ^17^ Institute of Social and Preventive Medicine University of Bern Bern Switzerland

**Keywords:** opportunistic infections, *Pneumocystis jirovecii* pneumonia, prophylaxis

## Abstract

**Introduction:**

Since the beginning of the HIV epidemic in resource‐rich countries, *Pneumocystis jirovecii* pneumonia (PjP) is one of the most frequent opportunistic AIDS‐defining infections. The Collaboration of Observational HIV Epidemiological Research Europe (COHERE) has shown that primary *Pneumocystis jirovecii* Pneumonia (PjP) prophylaxis can be safely withdrawn in patients with CD4 counts of 100 to 200 cells/µL if plasma HIV‐RNA is suppressed on combination antiretroviral therapy. Whether this holds true for secondary prophylaxis is not known, and this has proved difficult to determine due to the much lower population at risk.

**Methods:**

We estimated the incidence of secondary PjP by including patient data collected from 1998 to 2015 from the COHERE cohort collaboration according to time‐updated CD4 counts, HIV‐RNA and use of PjP prophylaxis in persons >16 years of age. We fitted a Poisson generalized additive model in which the smoothed effect of CD4 was modelled by a restricted cubic spline, and HIV‐RNA was stratified as low (<400), medium (400 to 10,000) or high (>10,000copies/mL).

**Results:**

There were 373 recurrences of PjP during 74,295 person‐years (py) in 10,476 patients. The PjP incidence in the different plasma HIV‐RNA strata differed significantly and was lowest in the low stratum. For patients off prophylaxis with CD4 counts between 100 and 200 cells/µL and HIV‐RNA below 400 copies/mL, the incidence of recurrent PjP was 3.9 (95% CI: 2.0 to 5.8) per 1000 py, not significantly different from patients on prophylaxis in the same stratum (1.9, 95% CI: 0.1 to 3.7).

**Conclusions:**

HIV viraemia importantly affects the risk of recurrent PjP. In virologically suppressed patients on ART with CD4 counts of 100 to 200/µL, the incidence of PjP off prophylaxis is below 10/1000 py. Secondary PjP prophylaxis may be safely withheld in such patients. While European guidelines recommend discontinuing secondary PjP prophylaxis only if CD4 counts rise above 200 cells/mL, the latest US Guidelines consider secondary prophylaxis discontinuation even in patients with a CD4 count above 100 cells/µL and suppressed viral load. Our results strengthen and support this US recommendation.

## 1 Introduction

Since the beginning of the HIV epidemic in resource‐rich countries, *Pneumocystis jirovecii* pneumonia (PjP) is one of the most frequent opportunistic AIDS‐defining infections [1]. PjP occurs predominantly in people living with HIV (PLWH) with marked immunodeficiency with CD4 positive lymphocytes in peripheral blood (CD4 count) of less than 200 cells/µL, or less than 14 % of total lymphocytes [2]. In the era before potent antiretroviral therapy (ART) this led to the recommendation of providing life‐long primary chemoprophylaxis against PjP in all PLWH with a CD4 count below this threshold. While PjP incidence decreased substantially in the era of ART it still occurs in patients presenting late during their HIV infection, in those lost to care, those non‐adherent or failing ART [3, 4]. The risk of recurrence of PjP after successful treatment of a first episode was shown to be particularly high [5, 6]. This may in part be due to genetic susceptibility for PjP [7]. Therefore, secondary prophylaxis or maintenance therapy was recommended for all patients having experienced PjP [8].

With the advent of potent ART, several studies have shown that primary and secondary PjP prophylaxis can be safely discontinued once the CD4 count rises to above 200 cells/µL [9, 10, 11, 12, 13, 14].

Within the large Collaboration of Observational HIV Epidemiological Research in Europe (COHERE), we have previously shown that HIV viraemia as measured by plasma HIV‐RNA is an additional important risk factor for PjP, independent of the CD4 count. Primary PjP prophylaxis may be safely withheld in patients on successful ART, with CD4 counts between 100 and 200 cells/µL [15]. In patients in this CD4 count stratum, with plasma HIV RNA below 400 copies/ML and off prophylaxis the incidence of primary PjP was 12 (95% CI 2 to 45) events per 1000 person‐years (py). A more recent analysis pointed towards safety of discontinuing prophylaxis even in all patients that achieve undectable plasma HIV‐RNA levels [16]. Similar findings were reported by smaller cohorts [17, 18, 19], a randomized trial [20] and in two systematic reviews [21, 22]. While the combined results of discontinuation of primary prophylaxis in the patients of interest indicated an incidence with a 95% confidence interval upper boundary below 10/1000 py, the data on discontinuation of secondary prophylaxis remained inconclusive [19, 20, 22]. Nevertheless, in recent years many physicians stopped prescribing PjP prophylaxis in patients on successful ART, even with low CD4 counts [23].

We evaluated the risk of secondary PjP in patients on and off prophylaxis at different levels of CD4 count and plasma HIV‐RNA based on a large database with long follow‐up.

## 2 Methods

### 2.1 Setting

COHERE in EuroCoord was a collaborative group of adult, pediatric, and mother/child HIV cohorts across Europe. The collaboration allows comparisons across age categories and provides a mechanism to rapidly compile datasets to address novel research questions that cannot be studied adequately in individual cohorts (www.eurocoord.net, www.cohere.org) [24]. Data were extracted from the 2015 merger of COHERE that included 23 European cohorts, with information on patient characteristics (age, sex and transmission category), use of ART (type of regimen and dates of start and discontinuation), CD4 cell counts and plasma HIV‐RNA over time and their dates, AIDS‐defining conditions and indicator variables for drop‐out or death.

### 2.2 Analysis

The results are reported as median and interquartile ranges for the quantitative variables, whereas those for qualitative variables are expressed as absolute frequency along with the associated percentage. The Kruskal–Wallis test was used for continuous and Fisher’s exact test for categorical variables.

We calculated the incidence rate (events per 1000py) stratified by current use of PjP‐prophylaxis and by plasma HIV‐RNA levels (<400, 400 to 10,000 copies/mL and >10,000 copies/mL). Poisson regression with a log link function was used to analyze associations between the incidence of PjP diagnosis, dependent on the use of secondary prophylaxis. Follow‐up started at the first visit at which the inclusion criteria were met (16 years of age or over, began follow‐up in their cohort after 1998, and a previous PjP diagnosis). As potential risk factors, we included gender (reference male), age, probable mode of HIV transmission (with categories men having sex with men (MSM) (reference), intravenous drug use (IDU), heterosexual and “other”), current HIV RNA level, current CD4 count and the contributing cohort. We fitted restricted cubic splines to model the smoothed effect of changing CD4 count over time using general additive Poisson models, calculated with sandwich‐type standard errors to adjust for patients contributing multiple periods of follow‐up time. These spline models enable the visualization of the incidence trajectory for continuous CD4 counts in a given viral load stratum. We varied the number of knots used for the splines to investigate the goodness of fit and to avoid overfitting.

The level of missingness for baseline and time‐varying variables was considered negligible (<10%), and therefore we did not resort to using formal missing data methods in the analysis. Last observation carried forward was used to impute missing post‐baseline CD4 and RNA measurements.

All analyses were carried out with R version 3.2.4, using the function “gam” in package “*mgcv*” to fit the general additive models [25, 26]. Throughout we considered a level of 0.05 as statistically significant.

### 2.3 Ethical approval

Ethical approval was applied for and granted for the research from the appropriate body in the host country of the cohort contributing the data to COHERE.

### 2.4 Patient consent

Patient consent was obtained by each of the cohorts as defined locally as a pre‐requisite for sharing information with the COHERE collaboration.

## 3 Results

There were 10,476 patients with PjP and a follow‐up time of 74,295 py at risk for secondary PjP. The overall incidence was 5.0 [4.5, 5.6] per 1000 py for 373 secondary PjP events occurring in the period 1998 to 2015. Patient characteristics for the total population and for those who did or did not develop secondary PjP are shown in Table 1. Those who developed secondary PjP were more likely to be female and infected by intravenous drug use. They were also younger and had lower CD4 counts and higher plasma HIV RNA at baseline.

**Table 1:** Characteristics of patients at baseline for those with and without a secondary PjP diagnosis during follow‐up

	Overall	No secondary PjP diagnosis	Secondary PjP diagnosis	*p*‐value
Number of patients	10476	10103	373	–
Female (%)	2082 (19.9%)	1987 (19.7%)	95 (25.5%)	<0.001
Age at baseline (median [IQR])	40 [35, 47]	40 [35, 48]	38 [34, 44]	<0.001
HIV transmission mode (%)				
MSM	4144 (39.6)	4020 (39.8%)	124 (33.2%)	<0.001
Heterosexual	3544 (33.8)	3429 (33.9%)	115 (30.8%)	
IDU	1390 (13.3)	1309 (13.0%)	81 (21.7%)	
Other, unknown	394 (3.4)	373 (3.7)	21 (5.6%)	
Missing	1004 (9.6)	972 (9.6%)	32 (8.6%)	
CD4 count/µL (median [IQR])				
Baseline	80 [24, 220]	80 [25, 221]	64 [22, 190]	0.07
At final follow‐up visit	–	415 [220, 628]	100 [29, 270]	<0.001
Plasma HIV‐RNA/mL (median [IQR])				
Baseline	71882 [500, 308000]	70000 [500, 306000]	101000 [11500, 351064]	<0.001
At final follow‐up visit	–	49 [22, 79]	24400 [155, 210000]	<0.001
Follow‐up time in years per patient (median [IQR])	–	6.5 [2.7, 11.5]	1.9 [0.6, 3.9]	<0.001
Percentage of follow‐up on ART (median [IQR])	–	96% [65%, 100%]	99% [64%, 100%]	0.1

ART, antiretroviral therapy; IDU, intravenous drug use; IQR, interquartile range; MSM, men who have sex with men; PjP, pneumocystis jirovecii pneumonia.

**Figure 1:**
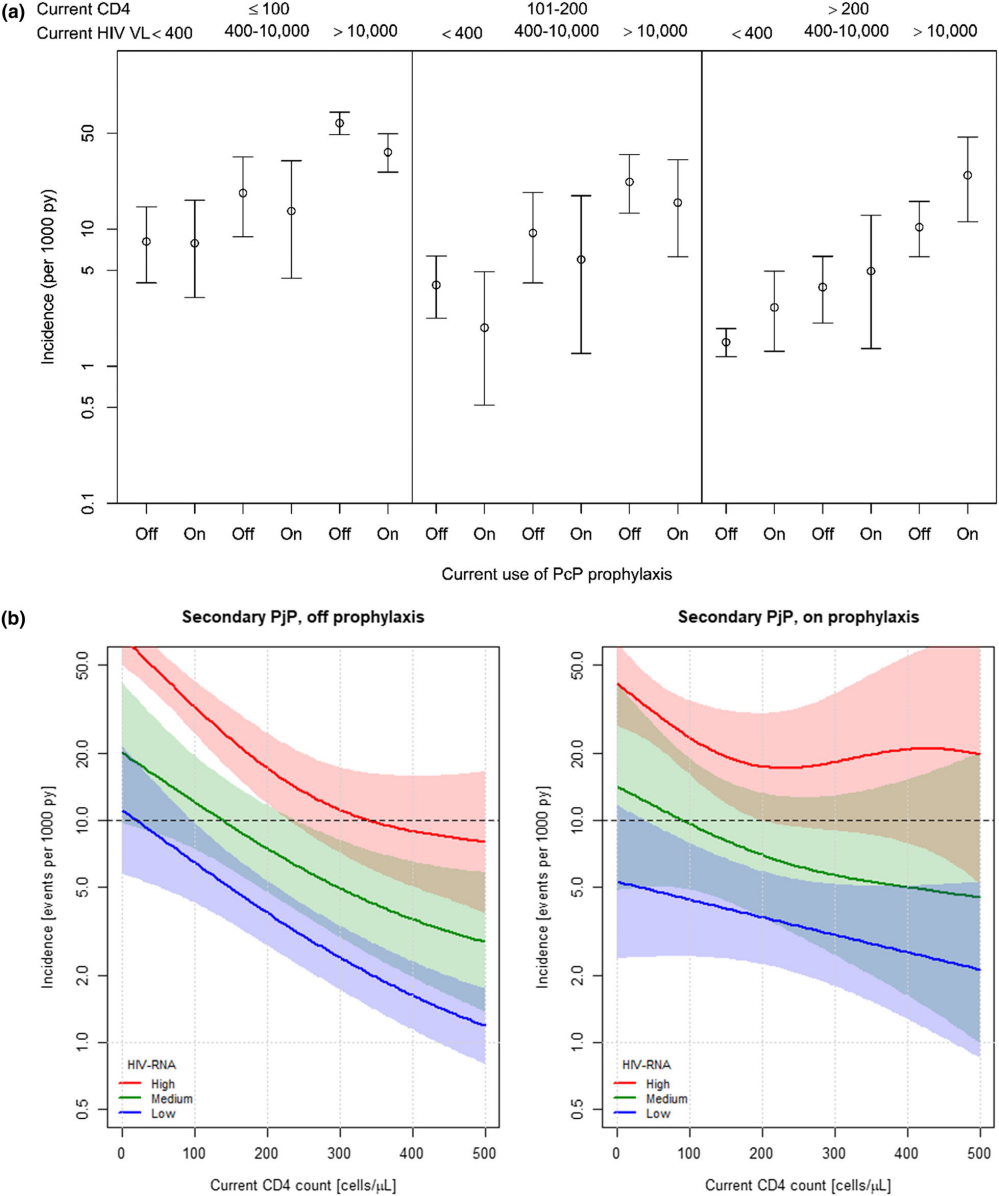
Incidence of secondary Pneumocystis Pneumonia (PjP). **(a)** Stratified by CD4 count (cells/µL) and plasma HIV‐RNA (copies/mL) levels for those on or off PjP prophylaxis; point estimates indicated by circles with 95% confidence intervals as vertical error bars. **(b)** Stratified plasma HIV‐RNA levels from those off (left panel) and on (right panel) PjP prophylaxis. Plasma HIV‐RNA levels: High >10,000, Medium 400‐10,000, Low <400 copies/mL. From the fitted Poisson general additive model for a 35 year old, male, MSM with 95% confidence intervals shown shaded in the respective colour. HIV, human immunodeficiency viruses; HIV‐RNA, human immunodeficiency viruses (HIV) Ribonucleic acid; MSM, men who have sex with men; PjP, *Pneumocystis jirovecii* pneumonia; Py, person years; Py, person years; RNA, Ribonucleic acid; VL, Viral load

Figure 1 and Table 2 depict the incidence in patients at risk of secondary PjP. The incidence of recurrent PjP in the low HIV‐RNA stratum with CD4 counts 100 to 200 cells/µL for patients off secondary prophylaxis was estimated to be 3.9 (95% CI: 2.0 to 5.8) events per 1000 py (Figure 1a), as compared to 1.9 (95% CI 0.1 to 3.7; *p* = 0.1) on prophylaxis. In patients with suppressed viral load, we could exclude an incidence of more than 10/1000py for those with current CD4 counts of at least 95 cells/ µL at a 95% confidence level.

**Table 2:** Incidence of secondary Pneumocystis Pneumonia (PjP) stratified by CD4 count (cells/µL) and plasma HIV‐RNA (copies/mL) and being on or off prophylaxis

CD4 strata	RNA strata	Secondary PjP prophylaxis	Number of events	Follow‐up time (1000 person years)	Incidence [95% CI]
<100	<400	No	11	1353.9	8.1 [3.3, 12.9]
<100	<400	Yes	7	887.0	7.9 [2.0, 13.7]
<100	400 to 10,000	No	10	545.6	18.3 [7.0, 29.7]
<100	400 to 10,000	Yes	5	369.2	13.5 [1.7, 25.4]
<100	>10,000	No	114	1920.4	59.3 [48.5, 70.3]
<100	>10,000	Yes	40	1098.5	36.4 [26.0, 49.6]
101 to 200	<400	No	16	4085.6	3.9 [2.0, 5.8]
101 to 200	<400	Yes	4	2096.7	1.9 [0.1, 3.7]
101 to 200	400 to 10,000	No	8	852.9	9.4 [2.9, 15.8]
101 to 200	400 to 10,000	Yes	3	499.4	6.0 [0.1, 12.8]
101 to 200	>10,000	No	18	814.0	22.1 [11.9, 32.3]
101 to 200	>10,000	Yes	7	448.3	15.6 [4.1, 27.2]
>200	<400	No	73	48776.8	1.5 [1.2, 1.8]
>200	<400	Yes	10	3728.5	2.7 [1.0, 4.3]
>200	400 to 10,000	No	14	3707.6	3.8 [1.8, 5.8]
>200	400 to 10,000	Yes	4	810.5	4.9 [0.1, 10.0]
>200	>10,000	No	20	1936.6	10.3 [5.8, 14.9]
>200	>10,000	Yes	9	363.9	24.7 [8.6, 40.9]

CI, confidence interval; HIV, human im.7munodeficiency viruses, PjP, pneumocystis pneumonia, RNA, ribonucleic acid.

Even in patients off prophylaxis in the CD4 count stratum below 100 cells/µL with suppressed viral load, the PjP incidence was low at 8.9/1000 py (95% CI 3.3 to 12.9). Figure 1b presents the estimates from the fitted Poisson model for a reference patient (35‐year old, male MSM) with a cubic spline smoother for the CD4 count, stratified by level of viral suppression (low, medium and high). According to this model, the upper boundary of the 95% confidence interval of PjP incidence increased to more than 20/1000 years in patients with very low CD4 counts (Figure 1b, left panel). As depicted in Figure 1b left panel, we found an incidence of <10/1000 py in patients with CD4 counts higher than 95 cells/µL at a 95% confidence level. There was a marginally significant higher risk for patients with transmission risk IDU compared to MSM as reference (RR 1.3, 95% CI 1.0 to 1.80 *p* = 0.05). The supplementary Figure presents the estimates from the fitted Poisson model for a 35‐year old, male IDU reference patient.

Viral replication was strongly associated with higher PjP incidence at every current CD4 count stratum (Table 2 and Figure 1) both for patients on and off prophylaxis. In fact, in patients with plasma HIV RNA above 10,000 c/mL we could not exclude an incidence of less than 10/1000 py for patients with current CD4 counts of more than 200 cells/μL in patients off or on prophylaxis (Figure 1b). The supplementary Table shows the estimates of incidence rate ratios from the fitted model for several risk factors.

## 4 Discussion

HIV viraemia is an independent major risk factor for secondary PjP, as we have previously demonstrated to be the case for primary PjP [15]. For patients on ART with suppressed viral load, the risk for secondary PjP is below 10/1000 py once their CD4 count has risen to above 95 cells/µL.

However, the risk for secondary PjP remains above 10/1000 py, even with CD4 counts of >200 cells/µL, in case of replicating HIV with a plasma HIV‐RNA level above 10,000 copies/mL.

Secondary PjP prophylaxis can therefore be safely discontinued in asymptomatic patients on ART with plasma viral loads of <400 copies/mL, a CD4 count above 100 cells/µL, and no other risk factors for PjP, such as additional immunosuppression or chronic lung disease. This would allow stopping or withholding secondary prophylaxis in most Western European patients with current CD4 counts between 100 and 200 cells/µL since viral suppression is achieved in more than 80% in patients on ART [27].

HIV replication has been shown to be an important, and CD4 count an independent, factor interfering with immuno‐competence. It has also been associated with a higher risk of tuberculosis [28], and with a lower response to vaccination against yellow fever [29], influenza [30] and hepatitis B [31].

Taking an upper 95% confidence limit for the incidence of PjP to be less than 10/1000 py as a threshold to safely withholding prophylaxis is somewhat arbitrary. However, in many of the pivotal studies defining the safety of discontinuation of PjP prophylaxis, the upper 95% confidence level of incidence was in this range, or even slightly higher [9, 10, 11, 12, 13, 14, 15]. Showing the smoothed effect of current CD4 count on incidence of PjP by fitting the spline models (Figure 1b) allows the treating physicians to define the safety margin themselves, based on our large observational database. The results of the models also show that within the stratum of CD4 counts of 100 to 200 cells/µL the incidence is not stable but is double at the lower end of this stratum compared to the higher end. Therefore, results relying on strata must be interpreted with caution.

Not being able to exclude an incidence of secondary PjP of less than 10/1000 py in patients with CD4 counts above 200 cells/µL in patients on virologically failing ART was intriguing, because CD4 counts in this range are generally considered protective for secondary PjP. This underlines the major influence of HIV replication on immune competence. Thus, our results even point towards a reconsideration of secondary prophylaxis against PjP in patients with established virological failure due to non‐adherence or non‐suppressive ART, even if they have a CD4 count above 200 cells/µL.

Furthermore, counterintuitively incidence seems to increase for those with “high” HIV‐RNA and CD4 >200 cells/μL when on prophylaxis (Figure 1b right‐hand side, red curve). While the apparent upwards trend could be due to overfitting of the spline curve, one has to consider that the follow‐up in this population is less than 5% of the follow‐up in PLWH with CD4 counts >200, and we did not find a statistically significant difference in incidence for these people relative to prophylaxis. Nevertheless, highly replicating HIV would imply non‐adherence to treatment or non‐suppressive ART. If non‐adherence to ART is the reason for replicating HIV, there may also have been non‐adherence to prophylaxis, which could explain an increased incidence for those on prophylaxis.

Current European and US Guidelines have been adapted recently to recommend discontinuation of primary PjP prophylaxis in patients with sustained viral suppression and CD4 counts above 100 cells/µL [32, 33]. While European guidelines recommend discontinuing secondary PjP prophylaxis only if CD4 counts rise above 200 cells/mL, the latest US Guidelines consider secondary prophylaxis discontinuation even in patients with a CD4 count above 100 cells/µL and suppressed viral load. Our results importantly strengthen and support this US recommendation.

Our study has the general limitations of observational analyses of multicohort collaborations. The analysis included data up to 2015, and therefore excludes more recent clinical practice and treatments. We did not verify single patient data for PjP events. However, the endpoint of PjP is clearly defined in all the collaborating cohorts. We used a plasma HIV‐RNA threshold of 400 copies/mL as a surrogate for viral suppression, whereas a viral load of less than 50 copies/mL more adequately defines viral suppression. By using the higher threshold our data about the safety of secondary prophylaxis discontinuation is consequently more conservative, and in practice, also allows discontinuing prophylaxis in patients with viral blips between 50 and 400 copies/mL.

In our model, patients were not required to have a plasma viral load <400 copies/mL for a certain time to be included in the low viraemia stratum. In clinical practice, it might however be advisable to discontinue prophylaxis once undetectable viraemia has been confirmed by more than a single measurement.

Lower age and IDU as HIV‐transmission risk were both associated with a somewhat higher risk of secondary PjP, which, as pointed out in other studies, might be an indication of lower ART adherence. In addition, co‐infections such as active hepatitis C could further compromise immune‐competence in IDUs.

Our recommendation may not apply to all geographical settings. Co‐trimoxazole prophylaxis has been shown to be associated with lower morbidity and mortality in patients with higher CD4 cell counts in Sub‐Saharan Africa, mainly due to its effectiveness in preventing infections other than PjP [34, 35]. Furthermore, our findings result from averaging data from many countries, and therefore individual settings may require a slightly more conservative approach.

## 5 Conclusions

In conclusion, in the absence of other risk factors, secondary PjP prophylaxis can be safely discontinued, or withheld, in patients who have both a CD4 count above 100 cells/µL and a suppressed viral load on ART. Conversely, it may be warranted to prescribe prophylaxis for viraemic patients with a viral load greater than 10,000 copies/mL, even those having a CD4 count above 200 cells/µL.

## Competing interest

None of the authors declared any relevant conflict of interest (refer to funding declaration below).

## Authors’ contributions

HF, MZ, DK and AA were responsible for the concept and methodology. AA and DK performed data curation and analysis. HF, MZ and JM were responsible for the supervision of the work. HF prepared the first draft of the manuscript. All authors were responsible for the interpretation of the analysis, reviewing and editing subsequent versions, and have read and approved the final manuscript.

## Supporting information


**Table S1**. Estimates of incidence rate ratios (IRR) for secondary *Pneumocystis jirovecii* pneumonia (PjP) from the fitted general additive model with a cubic spline smoother for CD4 count. In addition, there was a highly significant association of PjP with lower CD4 counts (*p* < 0.001). CI, Confidence interval; IDU, intravenous drug use; MSM, men who have sex with men
**Figure S1**. Incidence of secondary Pneumocystis Pneumonia (PjP) stratified plasma HIV‐RNA levels from those off (left panel) and on (right panel) PjP prophylaxis. Plasma HIV‐RNA levels: High >10,000, Medium 400‐10,000, Low <400 copies/mL. From the fitted Poisson general additive model for a 35‐year‐old male IDU patient with 95% confidence intervals shown shaded in the respective colour.
