# Supplementary material for: No need for secondary Pneumocystis jirovecii pneumonia prophylaxis in adult people living with HIV from Europe on ART with suppressed viraemia and a CD4 cell count greater than 100 cells/µL
Source: J Int AIDS Soc. 2021 Jun 12;24(6):e25726. doi: 10.1002/jia2.25726 (PMC8196713; doi:10.1002/jia2.25726)
Supplement: Supplementary file 1 — Table S1. Estimates of incidence rate ratios (IRR) for secondary Pneumocystis jirovecii pneumonia (PjP) from the fitted general additive model with a cubic spline smoother for CD4 count. In addition, there was a highly significant association of PjP with lower CD4 counts (p < 0.001). CI, Confidence interval; IDU, intravenous drug use; MSM, men who have sex with men Figure S1. Incidence of secondary Pneumocystis Pneumonia (PjP) stratified plasma HIV‐RNA levels from those off (left panel) and on (right panel) PjP prophylaxis. Plasma HIV‐RNA levels: High >10,000, Medium 400‐10,000, Low <400 copies/mL. From the fitted Poisson general additive model for a 35‐year‐old male IDU patient with 95% confidence intervals shown shaded in the respective colour. [file JIA2-24-e25726-s001.docx]

Supplementary material

Supplementary Table

Estimates of incidence rate ratios (IRR) for secondary *Pneumocystis jirovecii* pneumonia (PjP) from the fitted general additive model with a cubic spline smoother for CD4 count. In addition, there was a highly significant association of PjP with lower CD4 counts (p<0.001). MSM: Men who have sex with men; IDU: intravenous drug use, CI: Confidence interval

| Variable | IRR | 95% CI | p-value |
| --- | --- | --- | --- |
| Gender  Male  Female | Reference  1.23 | [0.98, 1.70] | 0.08 |
| Age (per year) | 0.99 | [0.97, 1.00] | 0.03 |
| Transmission Mode  ..MSM  ..IDU  ..Other  ..Heterosexual | Reference  1.34  1.20  0.96 | [1.00, 1.78]  [0.73, 1.95]  [0.72, 1.29] | 0.05  0.48  0.81 |
| PjP Prophylaxis  .. No  Yes | Reference  1.16 | [0.59, 2.27] | 0.67 |
| HIV plasma RNA (copies/mL)  ..Low (<400)  ..Medium (400-10’000)  High (>10’000) | Reference  2.02  5.11 | [1.19, 3.5]  [2.87, 9.12] | 0.01  <0.001 |
| *Interaction term*  RNA x PjP Prophylaxis  Low (<400) and No PjP Prophylaxis  ..Medium (400-10’000) and PjP Prophylaxis  ..High (>10’000) and PjP Prophylaxis | Reference  1.02  1.35 | [0.26, 4.07]  [0.35, 5.22] | 0.98  0.67 |

Supplementary Figure

Incidence of secondary Pneumocystis Pneumonia (PjP) stratified plasma HIV-RNA levels from those off (left panel) and on (right panel) PjP prophylaxis. Plasma HIV-RNA levels: High >10’000, Medium 400-10’000, Low <400 copies/mL. From the fitted Poisson general additive model for a 35-year-old male IDU patient with 95% confidence intervals shown shaded in the respective colour.


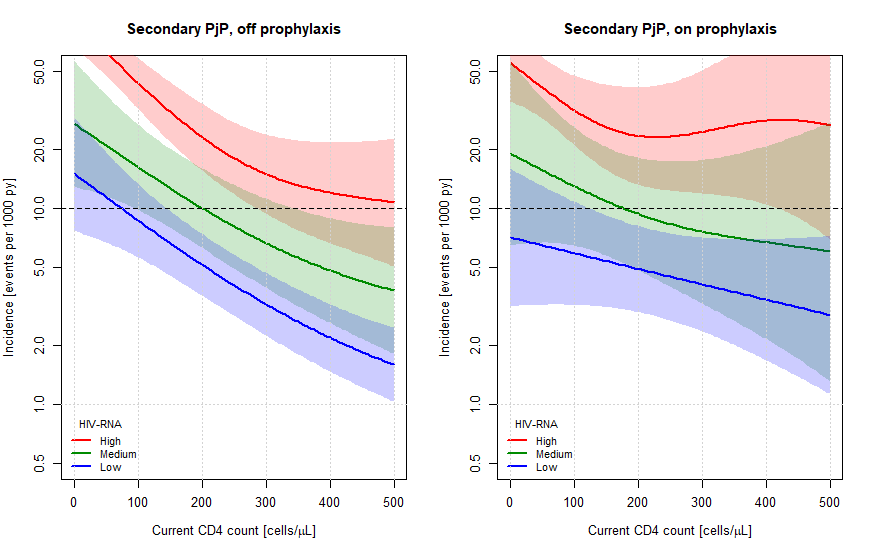


Notes: PjP *Pneumocystis jirovecii* pneumonia, HIV-RNA human immunodeficiency viruses (HIV) Ribonucleic acid, IDU: intravenous drug use, py person years, py person years.
